# Efficacy and Safety of Curcumin and *Curcuma longa* Extract in the Treatment of Arthritis: A Systematic Review and Meta-Analysis of Randomized Controlled Trial

**DOI:** 10.3389/fimmu.2022.891822

**Published:** 2022-07-22

**Authors:** Liuting Zeng, Tiejun Yang, Kailin Yang, Ganpeng Yu, Jun Li, Wang Xiang, Hua Chen

**Affiliations:** ^1^ Department of Rheumatology and Clinical Immunology, Peking Union Medical College Hospital, Chinese Academy of Medical Sciences & Peking Union Medical College, National Clinical Research Center for Dermatologic and Immunologic Diseases (NCRC-DID), Key Laboratory of Rheumatology and Clinical Immunology, Ministry of Education, Beijing, China; ^2^ Department of Orthopedics, People’s Hospital of Ningxiang City, Ningxiang City, China; ^3^ Key Laboratory of Hunan Province for Integrated Traditional Chinese and Western Medicine on Prevention and Treatment of Cardio-Cerebral Diseases, Hunan University of Chinese Medicine, Changsha City, China; ^4^ Department of Rheumatology, The First People's Hospital Changde City, Changde City, China

**Keywords:** curcumin, *Curcuma longa* extract, rheumatoid arthritis, ankylosing spondylitis, osteoarthritis, juvenile idiopathic arthritis, systematic review, meta-analysis

## Abstract

**Background:**

Modern pharmacological research found that the chemical components of *Curcuma longa L.* are mainly curcumin and turmeric volatile oil. Several recent randomized controlled trials (RCT) have shown that curcumin improves symptoms and inflammation in patients with arthritis.

**Methods:**

Pubmed, Cochran Library, CNKI, and other databases were searched to collect the randomized controlled trials (RCTs). Then, the risk of bias of RCTs were assessed and data of RCTs were extracted. Finally, RevMan 5.3 was utilized for meta-analysis.

**Results:**

Twenty-nine (29) RCTs involving 2396 participants and 5 types of arthritis were included. The arthritis included Ankylosing Spondylitis (AS), Rheumatoid Arthritis (RA), Osteoarthritis (OA), Juvenile idiopathic arthritis (JIA) and gout/hyperuricemia. Curcumin and Curcuma longa Extract were administered in doses ranging from 120 mg to 1500 mg for a duration of 4-36 weeks. In general, Curcumin and Curcuma longa Extract showed safety in all studies and improved the severity of inflammation and pain levels in these arthritis patients. However, more RCTs are needed in the future to elucidate the effect of Curcumin and Curcuma longa Extract supplementation in patients with arthritis, including RA, OA, AS and JIA.

**Conclusion:**

Curcumin and Curcuma longa Extract may improve symptoms and inflammation levels in people with arthritis. However, due to the low quality and small quantity of RCTs, the conclusions need to be interpreted carefully.

## 1 Introduction

Arthritis is a general term for various types of arthritic diseases, which are related to various factors such as degenerative diseases and autoimmunity. It is characterized by chronic inflammation of one or more joints, which usually causes pain and is often disabling. The main clinical symptoms are joint pain, swelling, stiffness, and limited mobility (1, 2). Epidemiology shows that arthritis is the most common in women, and the incidence of arthritis increases with age. Meanwhile, the prevalence of arthritis of different etiologies varies in the population (3, 4). Current research shows that there are more than 100 different forms of arthritis, with osteoarthritis (OA) and rheumatoid arthritis (RA) being the most common; other types mainly include arthritis associated with autoimmune diseases (5–7). Although these disorders have different etiologies, all of them are characterized by pain and limited mobility due to joint inflammation (7). At present, the drugs and non-drug methods for the treatment of arthritis are mainly related to the progression of joint pain and tissue joint inflammation, especially in the treatment of pain, the drugs are basically the same (8, 9). Among them, OA is a degenerative joint disease, the number of which is increasing with the aging of the population (10). According to the World Health Organization (WHO) survey, there are currently more than 400 million patients with osteoarthritis worldwide. In Asia, 1 in 6 people will develop OA at some point in their life. OA is more common in middle-aged and elderly people, and more women than men (11). Market research reports show that the OA therapeutics market was estimated at USD 6.8 billion in 2019 and is expected to reach USD 10.1 billion by 2024, growing at a CAGR of 8.1% from 2019 to 2024. The report shows that this increase is partly due to the rapid increase in the elderly and obese population and the consequent increase in the prevalence of OA (12–14). Rheumatoid arthritis (RA) is an autoimmune disease with erosive arthritis as the main symptom. The main symptoms are joint morning pain, swelling, pain and dysfunction. As a systemic inflammatory and destructive joint disease, the prevalence in the adult population worldwide is approximately 1-2% (15, 16). At present, RA is still difficult to cure, but standard diagnosis and treatment can achieve standard treatment. However, without regular treatment, it can lead to joint deformity and loss of function (17). Other types of arthritis are also associated with inflammation and pain, causing a huge burden on patients, but there is still no treatment for the underlying cause.

The main goal of current arthritis treatment is to reduce joint pain caused by joint inflammation, daily wear and tear of the joint, and muscle strain (18). Existing drugs for the treatment of arthritis are analgesics, steroids and non-steroidal anti-inflammatory drugs (NSAIDs), as well as biologically targeted drugs, which reduce symptoms such as severe pain and inflammation (19, 20). However, these drugs have a large number of side effects, which prevent them from providing sustained relief of disease symptoms and progression after long-term use. For example, the side effects of NSAIDs are severe gastrointestinal tract and insufficient pain relief after drug treatment, and biologically targeted drugs have immune disorders and adverse cardiovascular events (21–23). Therefore, the current treatment of arthritis has entered the stage of comprehensive management and treatment, and replacement therapy has gradually become an important part of the comprehensive management and treatment model (24, 25).

*Curcuma longa L.* is a potential alternative medicine for the treatment of arthritis, and they have been used as many ethnic medicines and gourmet condiments in several countries including China, Bangladesh, India and Pakistan (26). They have long been used as anti-inflammatory treatments in traditional Chinese medicine (TCM) and Ayurvedic medicines (27). The main components of turmeric are curcumin and demethoxycurcumin, bisdemethoxycurcumin and turmeric essential oil. Among them, curcumin is a natural compound, and current studies have shown that curcumin has good anti-inflammatory, immunosuppressive and anticancer properties (28–30). Evidence from multiple clinical trial studies suggests that curcumin can reduce the subjective experience of pain in patients with system-related disorders of muscle disease. Therefore, it is very important to systematically review the effects of *Curcuma longa L.* and curcumin in patients with arthritis.

## 2 Materials and Methods

### 2.1 Protocol

This systematic review and meta-analysis were conducted strictly in accordance with the protocol (CRD42022286421) and PRISMA-guidelines (see **Supplementary Materials**).

### 2.2 Literature Search Strategy

Web of Science, Cochrane Library, PubMed, The ClinicalTrials.gov, China Biology Medicine (CBM), VIP Database, China National Knowledge Infrastructure (CNKI), MEDLINE Complete, Wanfang Database, Embase were searched for RCTs related to Curcumin and Curcuma longa Extract in the treatment of arthritis. The search period is from the establishment of the database to Fib. 2022. The search strategy of Pubmed and Embase is shown in **Table S1** as an example.

### 2.3 Selection Criteria

(1) Participants: Patients diagnosed with any type of arthritis by recognized standards. (2) Intervention: the intervention of experimental group is curcumin, with no restrictions on dosage, dosage form, and usage; the intervention of control group can be non-curcumin interventions such as placebo and conventional therapy. (3) Outcomes: Efficacy indicators, inflammatory indicators, adverse events. (4) Study design: RCTs

### 2.4 Literature Screening, Data Extraction and Quality Assessment

Two researchers independently reviewed the literature according to Selection criteria, and conducted literature screening and data extraction. If there are differences, they should be resolved through consultation and discussion. The literature quality evaluation uses the risk bias assessment tool of the systematic reviewer manual recommended by the Cochrane Collaboration to evaluate the methodological quality of the included studies (31). The content of the assessment includes random allocation method, allocation concealment, whether blind method is used for participants, the completeness of the result data, whether there is selective reporting, and other biases.

### 2.5 Statistical Analysis

The RevMan 5.3 software recommended by the Cochrane Collaboration was used for meta-analysis. The risk ratio (RR) or mean difference (MD) and its 95% confidence interval (CI) are used as the efficacy and safety statistics. The χ2 test is used to evaluate the heterogeneity of the literature. P ≥ 0.1 or I 2 ≤ 50% means that the studies are homogeneous. The studies can be combined and analyzed using a fixed-effects model; otherwise, a random-effects model is used for analysis.

## 3 Results

### 3.1 Results of the Search

**Figure 1:**
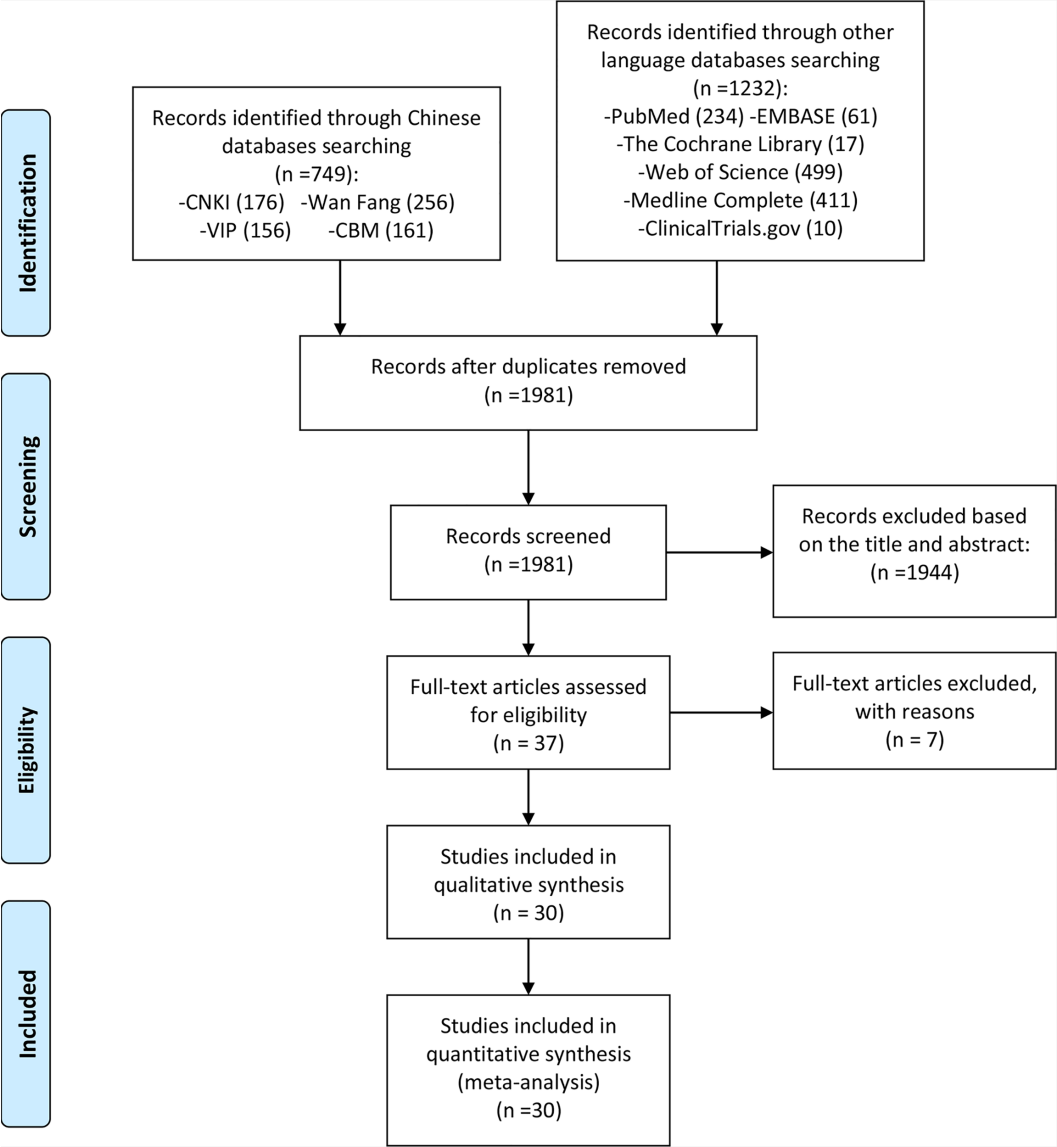
Flow diagram of clinical trials.

According to the search strategy, 1981 related papers were initially retrieved. After deduplication, reading the title and abstract, and the full text, 30 RCTs were finally included, while 7 records were excluded (32–38) (**Figure 1**).

### 3.2 Description of Included Trials

The included RCTs involved 5 types of arthritis: RA, AS, OA, Juvenile idiopathic arthritis (JIA) and gout/hyperuricemia; they also involved 11 countries: Iran, India, China, Australia, Belgium, Armenia, Indonesia, Thailand, Japan, Italy, Romania. The study size is between 20-200 participants. The drugs in the experimental group involved curcumin, curcuminoids and Curcuma longa Extract, and their types of preparation were different. 39, 40 and 41 used two different doses of curcumin intervention (high-dose group and low-dose group), so they are divided into 39 a (low-dose group), 39 b (high-dose group), 40 (low-dose group), 40 (high-dose group), 41 (low-dose group) and 41 (high-dose group). The interventions of Chandran et al. (42) were divided into Curcumin 500 mg and Curcumin 500 mg+diclofenac sodium 50 mg, so they were also divided into Chandran et al. (42) (Curcumin only) and Chandran et al. (42) (Curcumin+Diclofenac sodium). At the same time, their control group was divided into two groups, matching the two experimental groups. 43 include 2 experimental groups and 2 control groups; it was divided into 43 (Curcuma longa Extract v.s. placebo) and 43 (Curcuma longa Extract+Glucosamine v.s. Glucosamine). The details of study characteristics are presented in **Table 1**.

**Table 1:** The characteristics of the included studies.

Disease	RCTs	Country	Number of Participants (Female/male)		Intervention		Outcomes	Age (years)		Course of disease (years)		Duration
			Trial	Control	Trial	Control		Trial	Control	Trial	Control	
RA	Javadi et al. (44)	Iran	21/3	23/2	Curcumin nanomicelles 40mg Tid	wheat flour (placebo) 500mg Tid	DAS28, Tender joint count, Swollen joint count, ESR, adverse events	53.71 ± 2.75	56.28 ± 2.5	8.5 ± 9.08	7.36 ± 5.29	12 weeks
	Amalraj et al. (39)	India	8/16	7/5	Curcumin 250mg or 500mg	placebo	DAS28, ESR, CRP, Tender joint count, Swollen joint count, RF, adverse events	36.7 ± 10.7 (250mg); 38.3 ± 5.8 (500mg)	39.6 ± 8.8	–	–	12 weeks
	Jacob et al. (40)	India	7/9	5/3	Curcumin 250mg or 500mg	placebo	DAS28, ESR, CRP, RF	18-65	18-65	–	–	12 weeks
	Chandran et al. (42)	India	24/6	14/1	Curcumin 500 mg or Curcumin 500 mg+diclofenac sodium 50 mg	Diclofenac sodium 50 mg	DAS28, ESR, CRP, adverse events	47.8 ± 8.60 (Curcumin only); 47 ± 16.22 (combination)	48.87 ± 10.78	–	–	8 weeks
	Lin et al. (45)	China	32/32	33/31	Curcumin 100 mg Tid+Methotrexate 12.5mg once a week	Methotrexate 12.5mg once a week	DAS28, adverse events	48.39 ± 9.70	45 ± 10.31	9.43 ± 7.93	11.94 ± 8.02	12 weeks
	Pourhabibi-Zarandi et al. (46)	Iran	22/0	22/0	Curcumin 500 mg	placebo	ESR, CRP	50.68 ± 9.93	50.36 ± 9.70	9.77 ± 3.49	8.09 ± 3.13	8 weeks
OA	Hashemzadeh et al. 2007 (47)	Iran	29/7	31/4	Curcuminoids (SinaCurcumin™) 40mg	Placebo	WOMAC score, adverse events	54.11 ± 5.80	56.54 ± 5.77	4.51 ± 0.48	4.71 ± 0.48	6 weeks
		Italy	14/9	19/8	CartiJoint Forte (containing glucosamine hydrochloride (GH), chondroitin sulfate (CS) and Bio-Curcumin) + physical therapy	Placebo + physical therapy	VAS, WOMAC score, adverse events	71.3 ± 8.8	71.0 ± 8.0	6.8 ± 7.6	7.2 ± 6.0	8 weeks
	Haroyan et al. (48)	Armenia	62/5	65/3	Curcuminoids 999mg (CuraMed^®^ 1500mg)	Placebo	WOMAC score, ESR, CRP, adverse events	54.65 ± 8.84	56.04 ± 8.55	–	–	12 weeks
	Nakagawa et al. (49)	Japan	14/4	18/5	Curcumin 180 mg	Placebo	VAS, adverse events	71.9 ± 5.3	66.1 ± 7.2	–	–	8 weeks
	Madhu et al. (43)	India	41/19	42/18	Curcuma longa Extract 1000 mg or Curcuma longa Extract 1000 mg+ Glucosamine 1500 mg	Glucosamine 1500 mg or Placebo (Microcrystalline cellulose) 800mg	VAS, adverse events	56.63 ± 10.58; 58.17 ± 9.30	56.80 ± 7.99; 56.77 ± 9.98	–	–	6 weeks
	Srivastava et al. (50)	India	53/25	50/32	Curcuma longa Extract 500 mg+Diclofenac 50 mg	Placebo 500mg+Diclofenac 50 mg	VAS, WOMAC score, adverse events	50.23 ± 8.08	50.27 ± 8.63	–	–	16 weeks
	Khanna et al. (51)	India	42/38		Curcumagalactomannosides 400mg + glucosamine hydrochloride 500mg	Chondroitin sulphate 415 mg+ glucosamine hydrochloride 500 mg	VAS, WOMAC score	53.4 ± 6.64	51.5 ± 5.95	–	–	12 weeks
	Shep et al. (52)	India	48/21	45/25	Curcumin (BCM-95^®^) 1500 mg	Diclofenac sodium 100mg	VAS, KOOS, adverse events	53.09 ± 4.17	52.14 ± 3.76	0.62 ± 0.29	0.62 ± 0.26	4 weeks
	Thomas et al. (53)	India	16/19	21/16	Curcuminoids 500mg	Chondroitin sulphate 830 mg+ glucosamine hydrochloride 1000 mg	VAS, WOMAC score, adverse events	51.7 ± 5.52	52.3 ± 4.59	3.51 ± 0.56	3.38 ± 0.72	6 weeks
	Wang et al. (24)	Australia	18/18	21/13	Curcuma longa Extract 1000 mg	Placebo	VAS, WOMAC score, adverse events	61.3 ± 8.5	62.4 ± 8.8	–	–	12 weeks
	Pinsornsak et al. (54)	Thailand	62/13		Curcumin 1000 mg+diclofenac 75 mg	Diclofenac 75 mg	VAS	–	–	–	–	12 weeks
	Jamali et al. (55)	Iran	22/14	23/13	Curcumin ointment	Placebo (Vaseline ointment)	VAS, adverse events	68.86 ± 6.27	67.94 ± 6.72	7.22 ± 4.46	6.91 ± 4.68	6 weeks
	Kuptniratsaikul et al. (56)	Thailand	41/11	45/10	Curcuma longa Extract 2000 mg	Ibuprofen 800 mg	VAS, adverse events	61.4 ± 8.7	60.0 ± 8.4	1.59 ± 1.63	1.86 ± 2.2	6 weeks
	Kuptniratsaikul et al. (57)	Thailand	157/14	139/21	Curcuma longa Extract 1500 mg	Ibuprofen 1200 mg	WOMAC score, adverse events	60.3 ± 6.8	60.9 ± 6.9	4.28 ± 4.45	4.33 ± 4.31	4 weeks
	Panahi et al. (58)	Iran	14/5	17/4	Curcuminoid 1500mg	Placebo (inert starch)	VAS, WOMAC score, adverse events	57.32 ± 8.78	57.57 ± 9.05	–	–	6 weeks
	Lopresti et al. (59)	Australia	24/27	26/24	Curcuma longa Extract (Curcugen) 1000mg	Placebo	KOOS, adverse events	59.59 ± 6.57	57.92 ± 6.22	–	–	8 weeks
	Henrotin et al. (41)	Belgium	79/17	34/11	Curcuma longa Extract 280mg or 197mg	Placebo	VAS, KOOS, adverse events	60.9 ± 9.78; 61.4 ± 7.49	63.3 ± 7.69	7.41 ± 7.29; 6.6 ± 4.67	7.6 ± 9.3	12 weeks
	Kertia et al. (60)	Indonesia	24/15	29/12	Curcuminoid 90mg	Diclofenac sodium 90mg	COX-2	64.05 ± 8.83	64.56 ± 8.86	3.44 ± 2.72	3.36 ± 2.57	4 weeks
	Panahi et al. (66)	Iran	14/5	17/4	Curcuminoids (C3 complex^®^) 1500 mg	Placebo (inert starch)	SOD, GSH, MDA	57.32 ± 8.78	57.57 ± 9.05	–	–	6 weeks
	Gupte et al. (61)	India	11/6	23/2	Curcuma longa Extract 800mg	Ibuprofen + placebo	VAS, WOMAC score, adverse events	57 ± 7.5	54 ± 8	almost 1-11		6 weeks
AS	Ahmadi et al. (Ahmadi et al., 2020 (62)	Iran	12/0	12/0	Nanocurcumin	Placebo	Inflammatory factor	23-32	27-46	3-22	6-17	16 weeks
Juvenile oligoarthritis	Ailioaie and Ailioaie (63)	Romania	16 (not known)	16 (not known)	Protein Curcumin Complex 1800mg	Placebo	VAS, ACR Pedi30, ACR Pedi50, ACR Pedi70, ACR Pedi90, adverse events	8-16		–	–	36 weeks
	Ailioaie and Ailioaie (64)	Romania	28 (not known)	20 (not known)	Ultra Bioavailable Curcumin 1200mg	Placebo	JADAS-71	Mean: 13.8		–	–	24 weeks
Gout/hyperuricemia	Bupparenoo et al. (65)	Thailand	20 (8/12)	19 (7/12)	Curcumin 1000mg	Placebo	Serum uric acid, urine uric acid clearance, fasting plasma glucose, blood lipids, adverse events	55.5 ± 8.7	55.2 ± 13.0	–	–	8 weeks

### 3.3 Risk of Bias Assessments

#### 3.3.1 Selection Bias

Nine RCTs did not described the random sequence generation methods and were rated as unclear risk of bias (39, 40, 44, 49, 51, 54, 60, 63–65). Other studies have explained the method of generating random sequences, so they are assessed as low risk of bias.

63 and 39, 40, 45, 51, 60, 64, 66 did not specify whether to perform allocation concealment and were therefore assessed as unclear risk of bias. Other studies have described the method of allocation concealment, so they are assessed as low risk of bias.

#### 3.3.2 Performance Bias and Detection Bias

39, 40, 42, 49–51, 63 and 54, 58, 64, 65 stated that they used blinding, but did not describe how the blinding was performed, and was rated as unclear risk of bias. 45, 52, 53, 61 did not describe whether blinding was used, and its primary outcome is subjective evaluation index, which is easily affected by non-blinding, so it is assessed as a high risk of bias. 56 described only the blinding for outcome assessment and not the blinding of patients, it was rated as low risk for blinding of outcome assessment and high risk for bling of participants and personnel. 43 used blinding only on the participants, not the measurers, and thus it was rated as having low risk of bias in performance bias and having high risk of bias in detection bias. The other RCTs described the method blind implementation to patients and researchers or their outcomes are objective indicators, and are therefore considered to be a low risk of bias.

#### 3.3.3 Attrition Bias and Reporting Bias

The remaining RCTs did not have incomplete outcomes or the reasons for the missing and the number are balanced, hence they are therefore assessed as low risk of bias. Allioaie and Ailioaie 63 and Allioaie and Ailioaie 64 only have abstracts and no proposals for outcomes, so we do not have enough information to rate whether there is selective reporting, so it is assessed as unclear risk of bias. The other RCTs do not have selective reporting and are therefore considered to be a low risk of bias.

#### 3.3.4 Other Potential Bias

**Figure 2:**
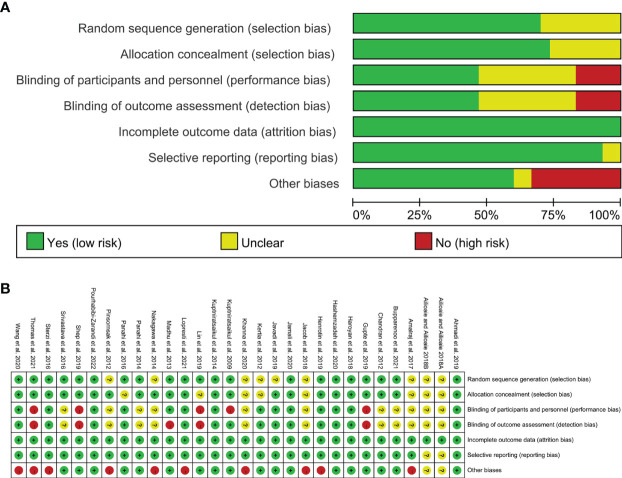
Risk of bias [**(A)** graph; **(B)** summary].

Ailioaie and Ailioaie 2018 only have abstracts, so we do not have enough information to rate whether there is selective reporting, so it is assessed as unclear risk of bias. 39–41, 49, 51, 53, 54, 59, 67, 68 claimed that authors have received funding from company that produces curcumin or Curcuma longa Extract; or that claimed that authors are the employees of company that produces curcumin or Curcuma longa Extract, hence they were rated as high risk of bias. Other sources of bias were not observed in 5 RCTs; therefore, the risks of other bias of the RCTs were low. (**Figure 2**)

### 3.4 The Outcomes for RA

#### 3.4.1 Efficacy Indicators

**Figure 3:**
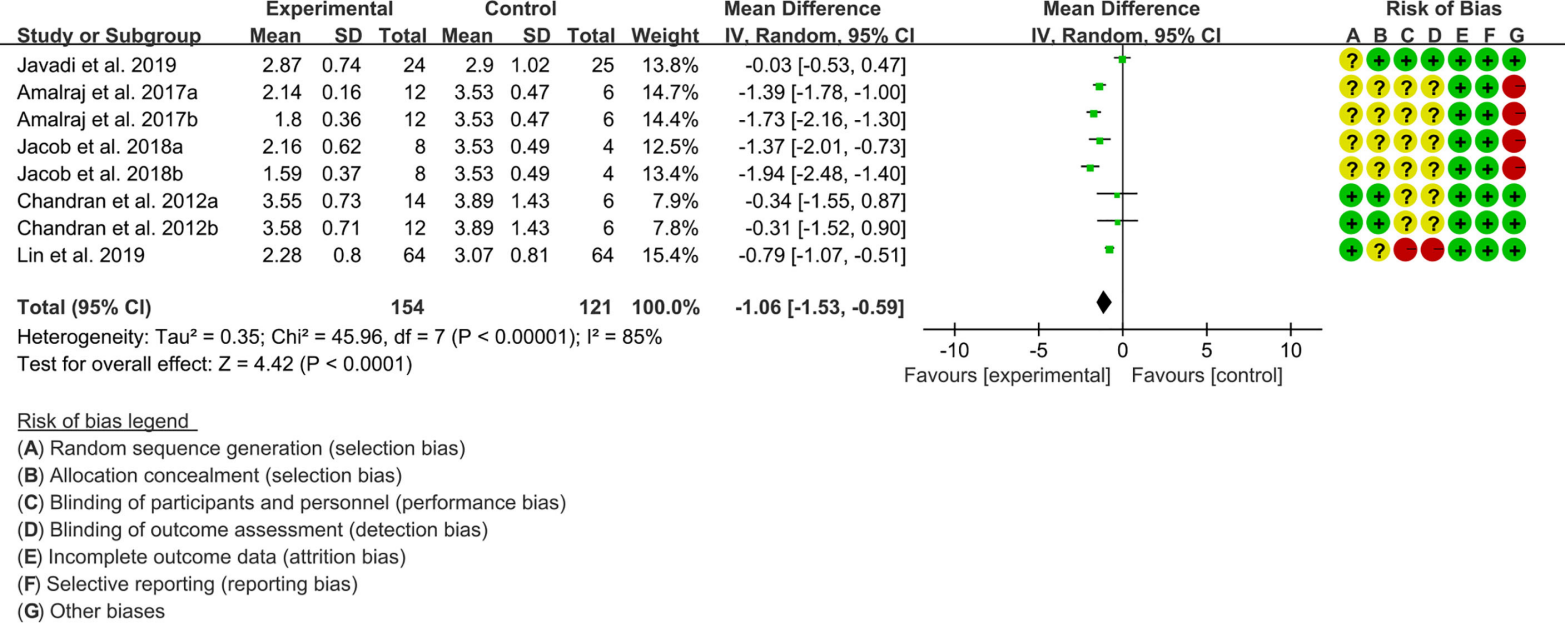
The results of DAS28.

**Figure 4:**
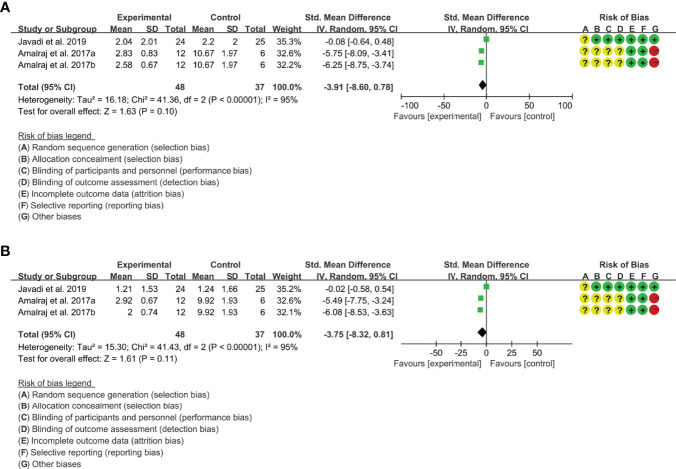
**(A)** Tender joint count; **(B)** Swollen joint count.

(1) DAS28: Five (5) RCTs reported the DAS28. The result of heterogeneity analysis was I2 = 85% and P<0.00001, which showed that there was statistical heterogeneity among the 5 studies, so the random effects model was used. The results of Meta analysis showed that there was a statistical difference between the experimental group and the control group (P<0.0001), which indicates that curcumin may decrease DAS28 [WMD -1.06 (-1.53, -0.59)] (**Figure 3**).

(2) tender joint count: Two (2) RCTs reported the tender joint count. The result of heterogeneity analysis was I2 = 95% and P<0.00001, which showed that there was statistical heterogeneity among the 2 studies, so the random effects model was used. The results of Meta analysis showed that the difference between the experimental group and control group is of no statistical significance [SMD -3.91 (-8.60, 0.78), P=0.10] (**Figure 4A**).

(3) swollen joint count: Two (2) RCTs reported the swollen joint count. The result of heterogeneity analysis was I2 = 95% and P<0.00001, which showed that there was statistical heterogeneity among the 2 studies, so the random effects model was used. The results of Meta analysis showed that the difference between the experimental group and control group is of no statistical significance [SMD -3.75 (-8.32, 0.81), P=0.11] (**Figure 4B**).

#### 3.4.2 Inflammatory Indicator

**Figure 5:**
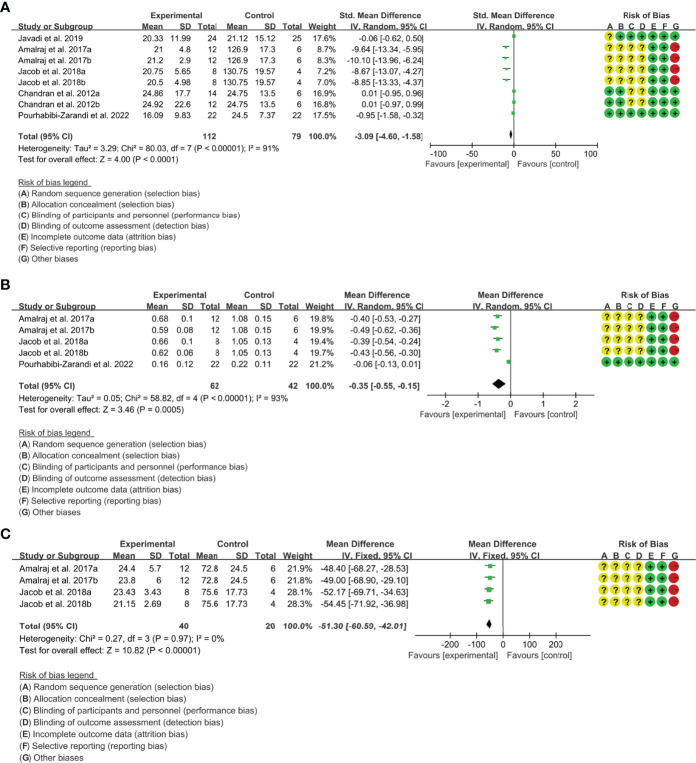
The results of ESR **(A)**, CRP **(B)** and RF **(C)**.

(1) ESR: Five (5) RCTs reported the ESR. The result of heterogeneity analysis was I2 = 91% and P<0.00001, which showed that there was statistical heterogeneity among the 5 studies, so the random effects model was used. The results of Meta analysis showed that there was a statistical difference between the experimental group and the control group (P<0.0001), which indicates that curcumin may decrease ESR [SMD -3.09 (-4.60, -1.58)] (**Figure 5A**).

**(**2) CRP: Four (4) RCTs reported the CRP. Since Chandran and Goel (42) differed in baseline CRP, the endpoint results were not comparable and the data were excluded. The result of heterogeneity analysis was I2 = 93% and P<0.00001, which showed that there was statistical heterogeneity among the 4 studies, so the random effects model was used. The results of Meta analysis showed that there was a statistical difference between the experimental group and the control group (P=0.0005), which indicates that curcumin may decrease CRP [WMD -0.35 (-0.55, -0.15)] (**Figure 5B**).

(3) RF: Two (2) RCTs reported RF. The result of heterogeneity analysis was I2 = 0% and P=0.97, which showed that there was no statistical heterogeneity among the 2 studies, so the fixed effects model was used. The results of Meta analysis showed that there was a statistical difference between the experimental group and the control group (P<0.00001), which indicates that curcumin may decrease RF [WMD -51.30 (-60.59, -42.01)] (**Figure 5C**).

#### 3.4.3 Adverse Events

**Figure 6:**
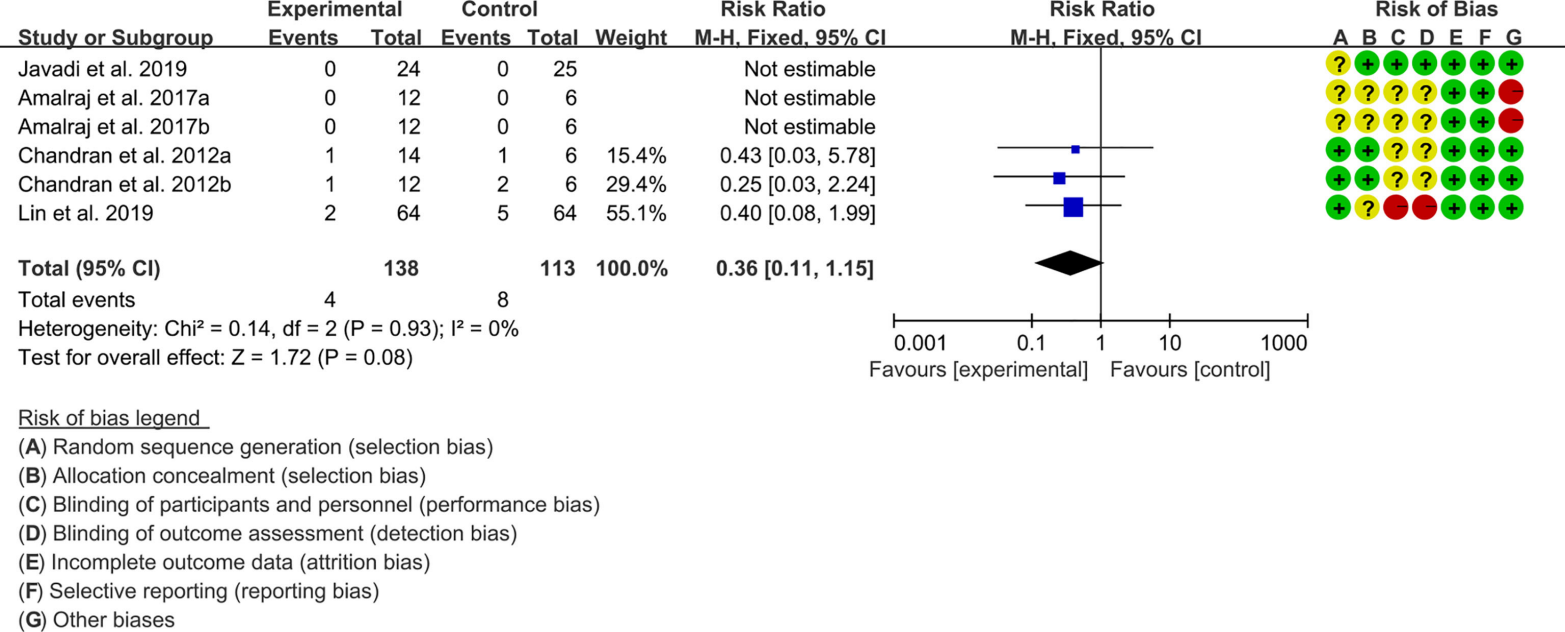
Adverse events.

Four (4) RCTs reported the adverse events. The result of heterogeneity analysis was I2 = 0% and P=0.93, which showed that there was no statistical heterogeneity among the 4 studies, so the fixed effects model was used. The results of Meta analysis showed that the difference between the experimental group and control group is of no statistical significance [RR 0.36 (0.11, 1.15), P=0.08] (**Figure 6**).

### 3.5 The Outcomes for OA

#### 3.5.1 Efficacy Indicators

The efficacy indicators include pain (include VAS and WOMAC-pain), physical function and stiffness. The efficacy indicators were divided into subgroups according to the intervention methods (**Table 2**).

**Table 2:** The subgroup analysis of OA efficacy indicators and adverse events.

Outcomes	Subgroup	Overall effect					Heterogeneity test			Figure
		MD	95%CI	P	I^2^ (%)	P	Statistical method	Studies (N)	Sample size (N)	
VAS	Curcuma longa Extract and curcumin (C.) v.s. placebo	-1.33	[-2.23, -0.43]	P=0.004	94	P<0.0001	Random	6	431	**Figure 7**
	C. v.s. NSAIDs	-0.07	[-0.32, 0.19]	P=0.62	0	P=0.54	Random	2	230	
	C.+ NSAIDs v.s. NSAIDs	-9.37	[-10.45, -8.28]	P<0.0001	–	–	Random	1	160	
	C. v.s. Articular cartilage nutrition drug	-2.71	[-5.91, 0.39]	P=0.09	98	P<0.0001	Random	2	204	
WOMAC-pain	C. v.s. placebo	-0.66	[-0.88, -0.43]	P<0.0001	34	P=0.21	Fixed	4	315	**Figure 8**
	C. v.s. NSAIDs	0.04	[-0.18, 0.25]	P=0.72	–	–	Fixed	1	331	
	C.+ NSAIDs v.s. NSAIDs	-4.1	[-4.65, -3.55]	P<0.0001	–	–	Fixed	1	160	
	C. v.s. Articular cartilage nutrition drug	-1.33	[-1.69, -0.98]	P<0.0001	1	P=0.32	Fixed	2	152	
WOMAC-physical function	C. v.s. placebo	-0.79	[-1.27, -0.31]	P=0.001	75	P=0.008	Random	4	315	**Figure 9**
	C. v.s. NSAIDs	0.07	[-0.14, 0.29]	p=0.51	–	–	Random	1	331	
	C.+ NSAIDs v.s. NSAIDs	-3.81	[-4.34, -3.29]	P<0.00001	–	–	Random	1	160	
	C. v.s. Articular cartilage nutrition drug	-3.1	[-4.34, -1.86]	P<0.00001	84	P=0.01	Random	2	152	
WOMAC-stiffness	C. v.s. placebo	-0.35	[-0.57, -0.12]	P=0.002	26	P=0.25	Fixed	4	315	**Figure 10**
	C. v.s. NSAIDs	0.05	[-0.17, 0.27]	P=0.65	–	–	Fixed	1	331	
	C.+ NSAIDs v.s. NSAIDs	-0.45	[-0.77, -0.14]	P=0.005	–	–	Fixed	1	160	
	C. v.s. Articular cartilage nutrition drug	-0.32	[-0.64, -0.00]	P=0.05	0	P=0.49	Fixed	2	152	
Adverse events	C. v.s. placebo	1.18	[0.71, 1.94]	P=0.52	25	P=0.25	Random	8	629	**Figure 13**
	C. v.s. NSAIDs	0.55	[0.34, 0.88]	p=0.01	70	P=0.03	Random	3	561	
	C.+ NSAIDs v.s. NSAIDs	0.53	[0.10, 2.79]	P=0.45	–	–	Random	1	160	
	C. v.s. Articular cartilage nutrition drug	0.58	[0.27, 1.24]	P=0.16	0	P=0.49	Random	2	158	

**Figure 7:**
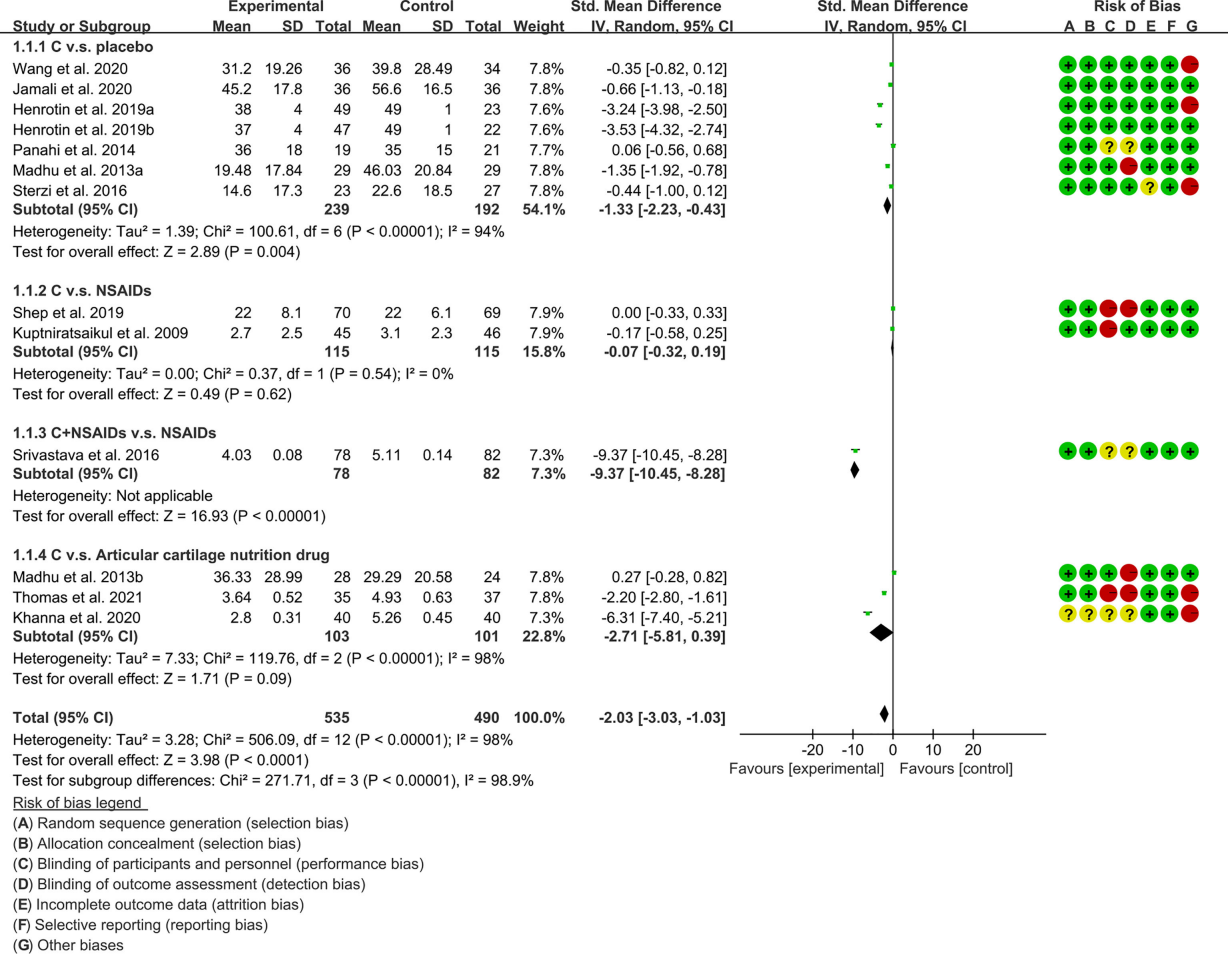
The results of VAS.

**Figure 8:**
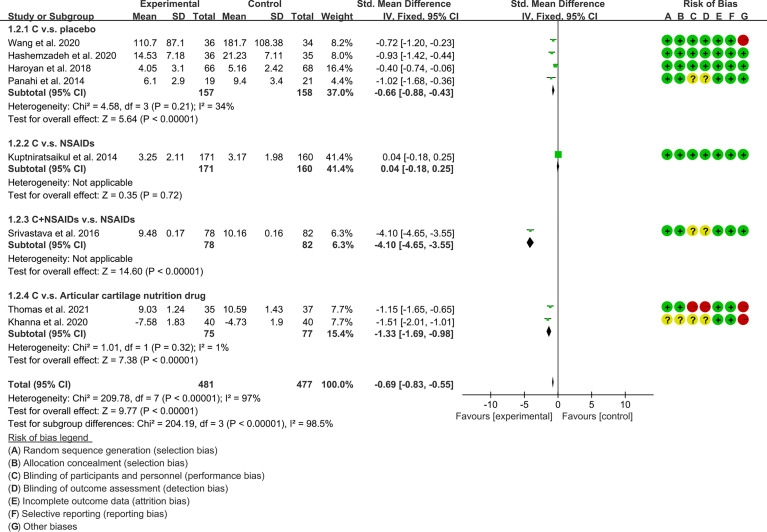
WOMAC-pain.

**Figure 9:**
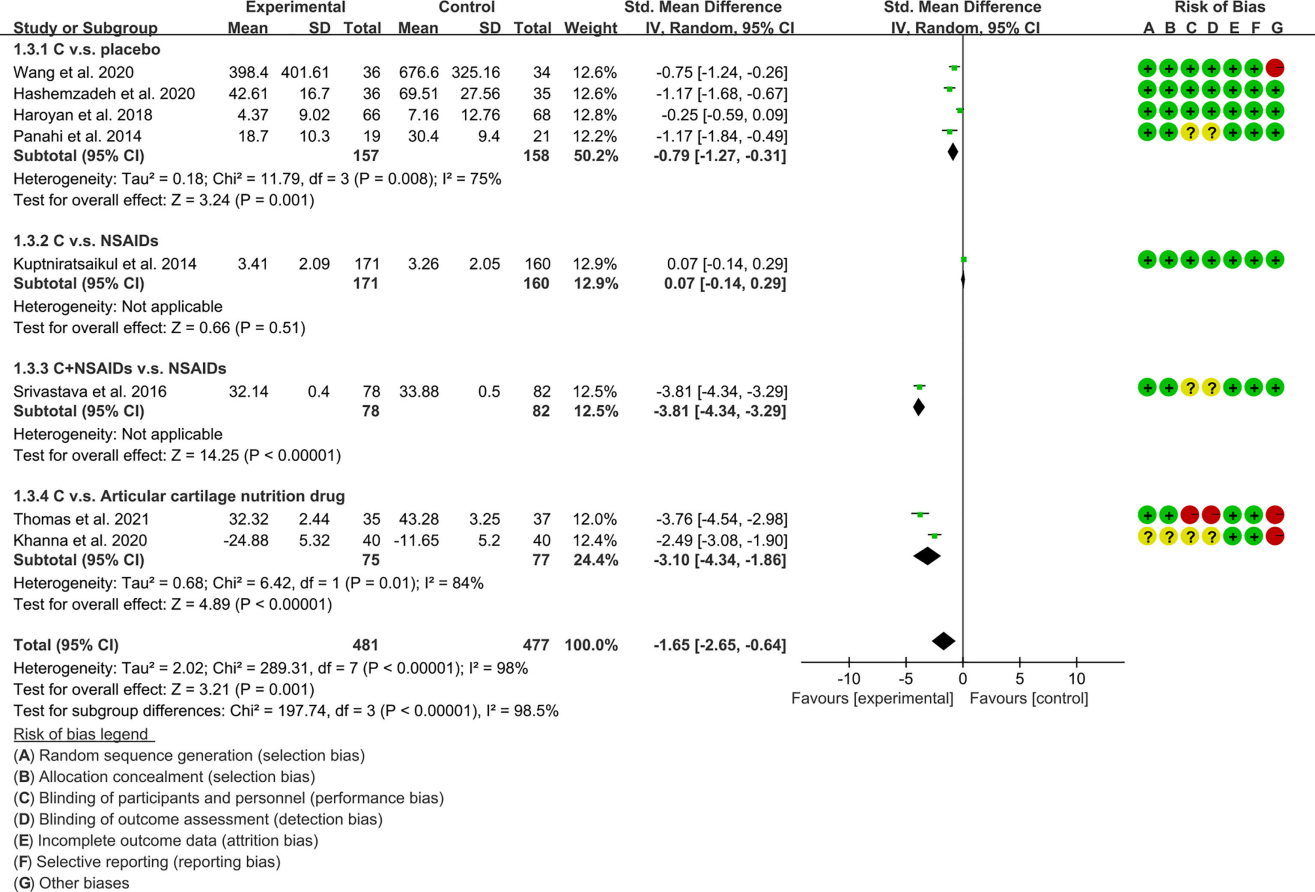
WOMAC-physical function.

**Figure 10:**
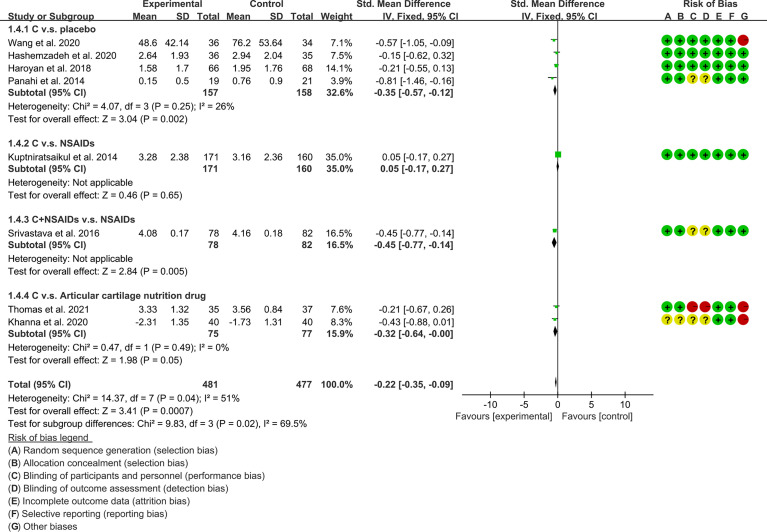
WOMAC -stiffness.

(1) Pain: The results of Meta analysis showed that there was a statistical difference between the experimental group and the control group (VAS: P<0.0001; WOMAC-pain: P<0.00001), which indicates that curcumin may decrease VAS and WOMAC-pain [VAS: SMD -2.03 (-3.03, -1.03); WOMAC-pain: SMD -0.69 (-0.83, -0.55)] (**Figures 7**, **8**).

(2) Physical function: The results of Meta analysis showed that there was a statistical difference between the experimental group and the control group (P=0.001), which indicates that curcumin may decrease WOMAC-physical function [SMD -1.65 (-2.65, -0.64)] (**Figure 9**).

(3) Stiffness: The results of Meta analysis showed that there was a statistical difference between the experimental group and the control group (P=0.0007), which indicates that curcumin may decrease WOMAC-stiffness [SMD -0.22 (-0.35, -0.09)] (**Figure 10**).

#### 3.5.2 Inflammatory Indicators

The inflammatory indicators include ESR, CRP and COX-2.

**Figure 11:**
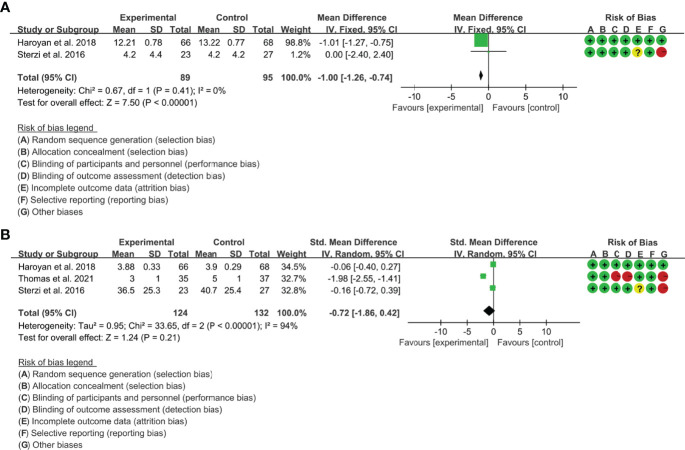
The results of ESR **(A)** and CRP **(B)**.

(1) ESR: Two (2) RCTs reported the ESR. The result of heterogeneity analysis was I2 = 0% and P=0.41, which showed that there was no statistical heterogeneity among the 2 studies, so the fixed effects model was used. The results of Meta analysis showed that there was a statistical difference between the experimental group and the control group (P<0.0001), which indicates that curcumin may decrease ESR [WMD -1.00 (-1.26, -0.74)] (**Figure 11A**).

(2) CRP: Three (3) RCTs reported the CRP. The result of heterogeneity analysis was I2 = 94% and P<0.00001, which showed that there was statistical heterogeneity among the 3 studies, so the random effects model was used. The results of Meta analysis showed that there was no a statistical difference between the experimental group and the control group [SMD -1.00 (-1.86, 0.42), P=0.21] (**Figure 11B**).

(3) COX-2 was only reported by 60, who found no significant difference in COX-2 between the diclofenac sodium group and the curcumin group (P=0.89).

#### 3.5.3 Oxidative Stress Related Outcomes

The indicators related to oxidative stress include SOD, GSH, and MDA.

**Figure 12:**
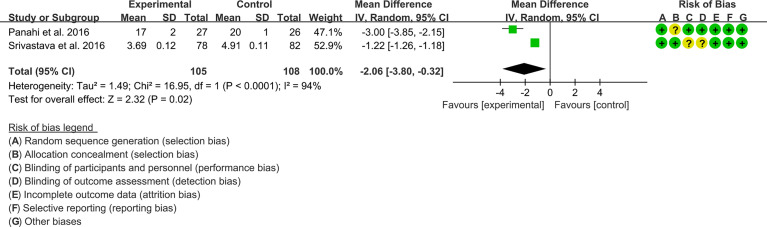
The results of MDA.

Two (2) RCTs reported MDA. The result of heterogeneity analysis was I2 = 94%, P<0.0001, which showed that there was statistical heterogeneity among the 2 studies, so the random effects model was used. The results of Meta analysis showed that there was a statistical difference between the experimental group and the control group (P=0.02), which indicates that curcumin may decrease MDA [WMD -2.06, (-3.80 to -0.32)]. (**Figure 12**).

Only Panahi et al., 2016 reported improvements in SOD and GSH and found higher serum SOD activity in the curcumin group compared to placebo (P<0.001). However, there was no statistically significant difference in GSH levels between the curcumin group and the placebo group (P=0.064).

#### 3.5.4 Adverse Events

**Figure 13:**
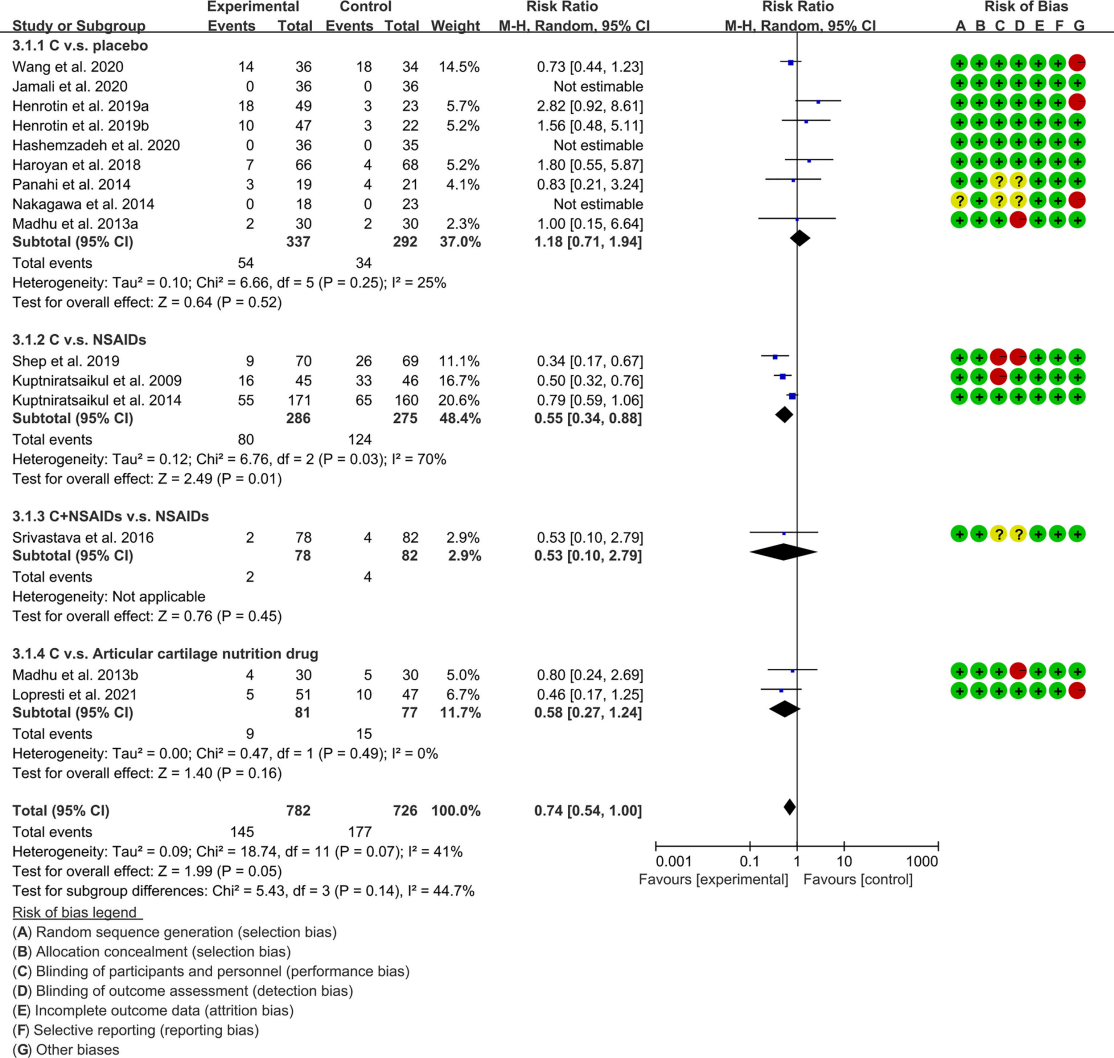
Adverse events.

The subgroup analysis results were shown in **Table 2**. 49, 53, 61 and 67 claimed that no serious adverse events were observed in either the experimental group or the control group. The summary result showed a borderline difference [RR 0.74, (0.54, 1.00), P=0.05; random effect model]. (**Figure 13**). If the study increases, it may be possible to find fewer adverse events with the addition of curcumin.

### 3.6 The Outcomes for AS

Only Ahmadi et al., 2020 (62) reported on AS. They reported that 12 patients received Nanocurcumin and 12 received Placebo. They found that the AS patients in the Nanocurcumin group had significantly increased Treg cells, increased IL-10 and TGF-β levels, and decreased IL-6 levels compared to control group. They also found that Nanocurcumin decreased the expressions of miR-17 and miR-27 and increased the expressions of miR-146a and FoxP3 (P<0.05).

### 3.7 The Outcomes for JIA

Two RCTs reported on JIA. Ailioaie et al., 63 enrolled 32 patients, ages 8-16, and randomly assigned them to curcumin group (receiving 600 mg three times a day) or placebo group for 9 months, while all patients received standard treat. Their study showed that compared with the control group, ACR Pedi30, ACR Pedi50, ACR Pedi70, and ACR Pedi90 were significantly improved in the curcumin group (P<0.05), and the addition of curcumin (1800mg/day) did not increase the incidence of adverse events. In another study, 48 children (mean age, 13.8 years) with extensive oligoarticular and polyarticular JIA were randomly assigned to experiment group (receiving curcumin 1,200 mg+blue laser) or control group (receiving placebo) for 6 months. They found that curcumin+blue laser reduced disease activity according to the Disease Activity Score (JADAS-71) and pain levels (0-10 cm VAS), it also increased their functional activities of daily living (CHAQ scores) compared to placebo (64).

### 3.8 The Outcomes for Gout/Hyperuricemia

Only 65 reported on hyperuricemia. They found that curcumin intervention tended to reduce serum uric acid compared with placebo, but the difference was not statistically significant (P=0.532). There were no significant differences in urine uric acid clearance, fasting plasma glucose, and blood lipids between the two groups (P>0.05). Compared with the placebo group, curcumin did not increase the incidence of adverse events (P>0.05), and the most common adverse event was diarrhea.

## 4 Discussion

**Figure 14:**
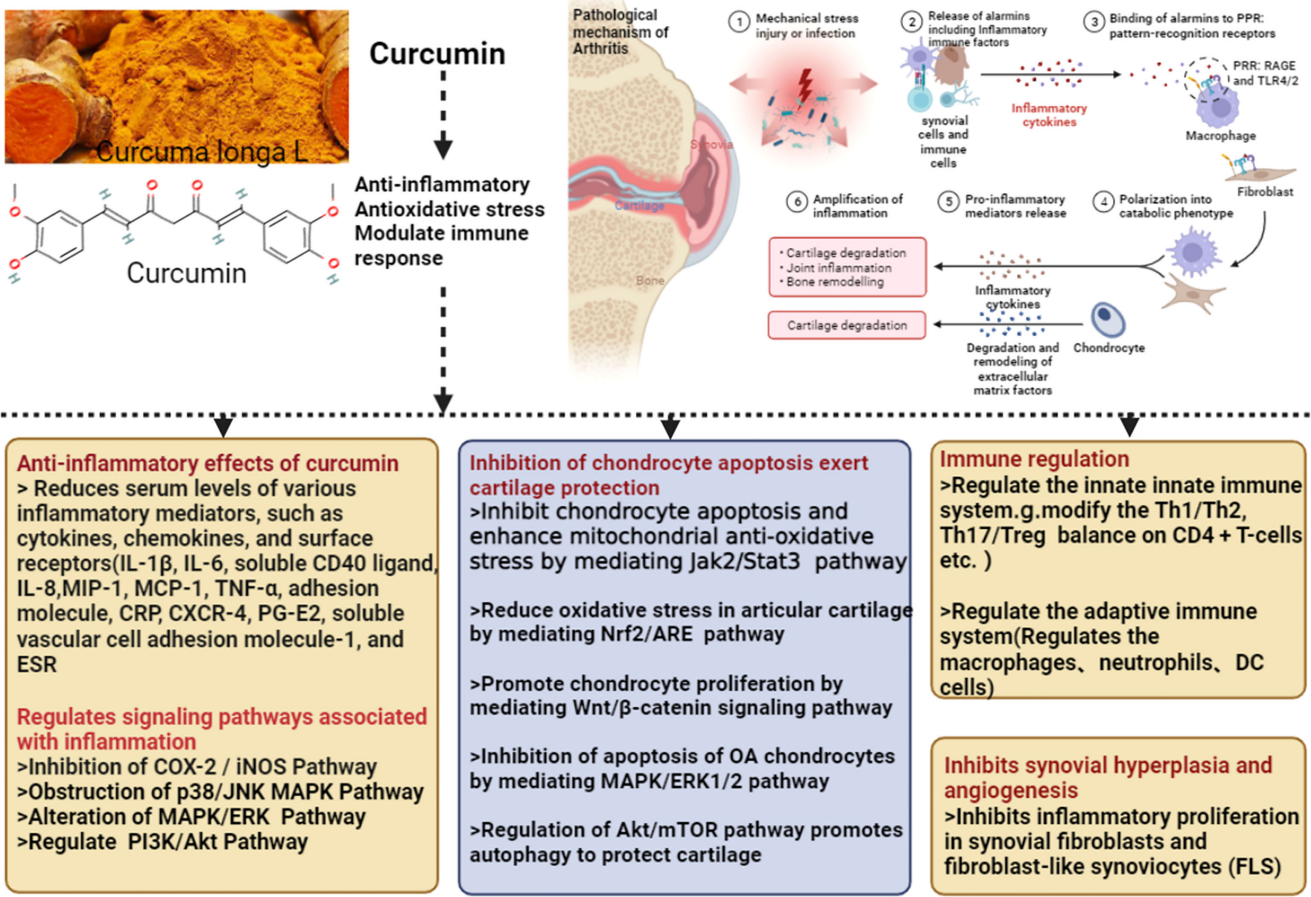
The mechanism of Curcumin and Curcuma longa Extract in the treatment of arthritis.

The mechanism of Curcumin and Curcuma longa Extract in the treatment of arthritis is shown in **Figure 14**.

### 4.1 Curcumin and *Curcuma longa* Extract for RA

This study systematically evaluated the clinical efficacy and safety of curcumin in the treatment of RA. The clinical efficacy indicators DAS28, ESR, CRP and RF were lower than those in the control group, indicating that curcumin may improve the symptoms of RA and inhibit the inflammatory response. For safety, the addition of curcumin may not increase the probability of adverse events.

Sun et al. constructed a model of destabilization of the medial meniscus (DMM) in mice. They found that curcumin can reduce the expression of IL-1β, interferon-γ (IFN-γ), IL17α, IL-18, TNF-α, (vascular cell adhesion molecule 1 (VCAM1), and inhibit the inflammatory response in mice (69). This is similar to the findings of this study. This meta-analysis shows that curcumin can reduce inflammation indicators (RF, ESR, CRP). Recent studies have shown that curcumin may inhibit osteoclast (OC) differentiation in RA patients by down-regulating the expression of RANK gene and related proteins (70). Curcumin combined with methotrexate can effectively improve the joint and systemic symptoms of RA patients, and can improve bone destruction, and has a significant intervention effect on the RANK/RANKL/OPG system (45). Curcumin can also inhibit the proliferation of fibroblast-like synovial cells (RA-FLS) and reduce the secretion of TNF-α and IL-6 in RA patients (71). Curcumin has a therapeutic effect on rats with type II collagen-induced arthritis (CIA). It can effectively inhibit macrophage-related inflammation and reduce the synovial homogenate of joints. It can also reduce the degradation of IκBα and the expression of COX-2 in RAW 264.7 cells (72). Xu et al. found that curcumin can reduce osteoclast production by inhibiting NF-κB signaling activation in RA (73). Curcumin can inhibit synovial angiogenesis in adjuvant arthritis (AA) rats. The mechanism may be related to reducing the expression of HIF-1α and down-regulating the expression of target genes VEGF and VEGFR, which may be one of the mechanisms of its treatment of RA (74).

### 4.2 Curcumin and *Curcuma longa* Extract for OA

The meta-analysis of OA found that: (1) Compared with placebo, Curcumin and Curcuma longa Extract may reduce pain, improve joint function, and improve joint stiffness; and the addition of Curcumin and Curcuma longa Extract did not increase adverse events. (2) Curcumin and Curcuma longa Extract and NSAIDs have similar effects in improving joint pain, function, and stiffness, but with a lower incidence of adverse events. However, when curcumin was used in combination with NSAIDs, it improved joint pain, function, and stiffness more than NSAIDs alone, without increasing the rate of adverse events. But, due to the small number of RCTs, definitive conclusions are difficult to draw. (3) Compared with articular cartilage nutritional drugs, Curcumin and Curcuma longa Extract may improve joint pain, joint function, and joint stiffness without increasing the incidence of adverse events. (4) Compared with the control group, Curcumin and Curcuma longa Extract could reduce ESR and MDA levels. For SOD, GSH and COX-2, no clear conclusions could be drawn due to the small number of RCTs.

OA is a common chronic disease that mainly affects the knee joint, resulting in joint pain and loss of function (10). Knee OA carries a high societal cost, but management options are few and not ideal (75). Current medical treatment options are limited to analgesics, intra-articular corticosteroids, and NSAIDs (76). Although they have some efficacy in relieving pain, they are associated with gastrointestinal, renal, and cardiovascular complications and are generally contraindicated in patients with comorbidities (77). *Curcuma longa L.* has a long history of medicinal use (78–80). Curcumin, the main and most pharmacologically active ingredient in *Curcuma longa L.*, is “generally recognized as safe” by the US FDA (81–83). Current *in vitro* and preclinical studies have demonstrated the potential of curcumin, Curcuma longa Extract, and other Curcuma longa Extract multi-herbal preparations to delay OA progression and relieve OA-related pain (84–86).

Compared to previous systematic reviews (86, 87), this study pooled the largest number of RCTs, including more evidence and relevant content. This improves the quality and evidence of this study to provide more realistic and precise effect sizes. In addition, we found that OA patients in the curcumin group may be less likely to initiate pain medication and more likely to discontinue their existing pain medication because of its efficacy and better safety profile compared to the NSAIDs group. A previous systematic review report on the effects of Curcuma longa Extract on chronic inflammatory diseases, including rheumatic diseases, showed no significant between-group differences in inflammatory markers between Curcuma longa Extract and placebo (88), however, our results found that Curcuma longa Extract could improve ESR in OA patients. Although Curcumin and Curcuma longa Extract are effective and safe for OA, these results are only from short-term studies (maximum follow-up of 16 weeks), but are expected to be effective and safe drugs, as most current OA drug treatments have poor safety profiles (89). Furthermore, the meta-analyses showed significant heterogeneity, which could be explained by study-level covariates such as BMI and age.

### 4.3 Curcumin and *Curcuma longa* Extract for AS

AS mainly affects the axial skeleton, sacroiliac joints, and peripheral joints, causing structural changes and dysfunction, and is a chronic autoimmune inflammatory disease (90–92). Chronic inflammation of the spine resulting from the progression of AS leads to the formation of new bone on the spine, ultimately resulting in spinal immobility and stiffness (93). Previous studies have suggested that the pathogenesis of AS may be related to bacterial infection and human leukocyte antigen B27 (HLA-B27), and recent studies also suggest that T lymphocytes may mediate AS (94, 95). This notion is supported by changes in peripheral blood (PB) CD4+ T cell frequency in patients with AS, including an increase in Th17 frequency with Th2 and a decrease in CD4+CD25+ regulatory T (Treg) cells (93, 95–97). Numerous studies have shown that patients with AS have a decreased Treg/Th17 ratio, suggesting that immune phenotype changes may be one of the pathogenesis of AS, and that regulating the balance of Treg/Th17 may reduce disease activity (96, 98, 99). Treg/Th17 functional balance is critical for the prevention of autoimmune and inflammatory diseases by preventing deleterious damage to the host and generating an effective immune response.

The current flow cytometry analysis of PB from AS patients showed that daily treatment with nanocurcumin for 4 months significantly increased the percentage of PB Treg cells compared to patients receiving placebo. Recent studies have found that curcumin can enhance Treg differentiation by increasing the expression of FoxP3 (94, 96). The results showed that FoxP3 gene expression was significantly increased in AS patients after nano-curcumin treatment, which confirmed the effect of nano-curcumin in enhancing Treg cells in these patients. In conclusion, this RCT study showed that administration of nanocurcumin (80 mg/kg bw/day) for 4 months increased the Treg population and the expression levels of FoxP3, TGF-β and IL-10, as well as inhibited the IL-6 cytokine level. Furthermore, nanocurcumin could effectively alter the expression of Treg-related miRNAs (decreased miR-17, miR-27 and increased miR-146a) during the follow-up of AS patients. More research is still needed in the future to further explore the exact biological process that curcumin modulates in AS patients. In addition, higher-quality multiple RCTs provide higher-quality evidence, thereby providing clinical value.

### 4.4 Curcumin and *Curcuma longa* Extract for JIA

The pathogenesis of JIA is related to a variety of factors, including genetic factors, immune responses, and environmental exposures. The pathogenesis of JIA is associated with aberrant activation of phagocytes (monocytes, macrophages, and neutrophils), suppression of Treg cells, hyperactivation of Th1 and Th17 cells, activation of NF-κB, and proinflammatory cytokines (IL-1, IL-6, IL-17, IL-18, IL-21, IL-22, IL-23, Interferon-γ [IFNγ] and TNF-α) (100–103). Currently, immunomodulatory drugs are the cornerstone of the treatment of JIA for these pathological processes of immune inflammation. Ailioaie et al., 63 included 32 children (ages 8-16 years) with JIA. They found that compared with the control group, ACR Pedi30, ACR Pedi50, ACR Pedi70, and ACR Pedi90 were significantly improved in the curcumin group (P<0.05), and the addition of curcumin (1800mg/day) did not increase the incidence of adverse events. In another study, 48 children (mean age, 13.8 years) with extensive oligoarticular and polyarticular JIA were randomly assigned to experiment group (receiving curcumin 1,200 mg+blue laser) or control group (receiving placebo) for 6 months. They found that curcumin+blue laser reduced disease activity according to the Disease Activity Score (JADAS-71) and pain levels (0-10 cm VAS), it also increased their functional activities of daily living (CHAQ scores) compared to placebo (64)

In the Miserocchi et al. (104) cohort study, 27 patients (age 17.4 ± 8.9 years) with oligoarticular JIA-associated uveitis were recruited and received curcumin (500 mg per day) and JIA standard of care for 12 months. Uveitis is a serious extra-articular complication of JIA. The severity of uveitis was assessed by slit-lamp examination and FC500 laser flare at baseline and 1, 3, 6, 9, and 12 months after curcumin initiation. A total of 22 patients (81%) had inactive uveitis at the end of the study. Five patients remained stable, and three developed uveitis flares. During the study period, curcumin supplements were well tolerated and no one experienced ocular side effects or allergic reactions [7]. Although the above studies have shown curcumin in JIA with considerable evidence of its clinical adjunctive value, more large-scale, randomized, placebo-controlled RCTs are needed in the future to confirm or revised the findings of these RCTs.

### 4.5 Curcumin and *Curcuma longa* Extract for Gout/Hyperuricemia

Hyperuricemia and gout is a metabolic abnormal syndrome caused by disturbance of purine metabolism. Because blood uric acid exceeds its saturation in blood or tissue fluid, sodium urate crystals are formed and deposited in the local joints, which induces local inflammation and tissue destruction symptoms (105, 106). The deposition of sodium urate crystals in the kidneys can cause acute kidney disease, chronic interstitial nephritis or kidney stones, which is called uric acid nephropathy. The common symptoms of hyperuricemia and gout are joint swelling and pain, starting from the feet, and then to the finger joints or wrists. Epidemiology shows that the number of patients with hyperuricemia and gout is increasing year by year worldwide, and it is expected to reach 1.42 billion in 2030 (107, 108). Patients with hyperuricemia and gout have poor compliance, with more than 50% of patients failing to follow doctor’s orders for examination or re-examination, and the number of hyperuricemia and gout patients is still increasing (109; 110). Curcumin has shown potential effects in hyperuricemia and gout (65). Basic studies have shown that curcumin can inhibit the degradation of IκBα, the activation of NF-κB signaling pathway, and the inflammatory genes downstream of NF-κB in monosodium urate-stimulated THP-1-derived macrophages (111). Curcumin protected THP-1 and RAW264.7 cells from monosodium urate-induced mitochondrial damage by preventing the reduction of mitochondrial membrane potential, reducing mitochondrial reactive oxygen species, and then inhibiting the activity of the NLRP3 inflammasome. Animal studies have also shown that intraperitoneal injection of curcumin attenuates monosodium urate crystal-induced paw and ankle swelling, inflammatory cell infiltration, and MPO activity in a mouse model of acute gout. These results were related to inhibition of IκBα degradation, phosphorylation levels of NF-κB subunits (p65 and p50) (111). *In vitro* and *in vivo* studies on hyperuricemia have shown that curcumin and its degradation products have xanthine oxidase inhibition (112–114) and uric acid production by inhibiting URAT1 (115). Therefore, the drug may be effective in reducing serum uric acid. In addition, curcumin has been reported to reduce serum uric acid levels in other diseases (non-alcoholic fatty liver disease (NAFLD) and diabetes mellitus) (116; 117).

### 4.6 The Safety of Curcumin and *Curcuma longa* Extract

According to RCTs reporting adverse events, Curcumin and Curcuma longa Extract did not increase the occurrence of adverse events. According to the report of Food and Agriculture Organization of the United Nations/World Health Organization (FAO/WHO) and European Food Safety Authority (EFSA), the acceptable daily intake (ADI) value of curcumin is 0-3 mg/kg; it is also approved by the US Food and Drug Administration as a botanical (79). According to the relevant safety and toxicity clinical trials, the acceptable dose of curcumin to obtain the maximum efficacy is 4-8 g/d. The dose of 8 g/d curcumin was shown to be safe in both phase I and II clinical trials (118), and it has been reported that curcumin up to 12 g/d is still tolerated by humans (119). Studies have shown that curcumin has no obvious sub-chronic toxicity damage after animal toxicity test, and has no potential mutagenic or teratogenic effects (120). For example, Krishnaraju et al. conducted toxicological evaluations on the safety of demethyl curcumin (DC) through acute oral administration, acute skin, primary skin and eye irritation, and dose-dependent 90-day subchronic toxicity studies. They found that the acute oral median lethal dose (LDso) of DC in female SD rats was >5 000 mg/kg, and the acute dermal LD50 was >2000 mg/kg, and no weight change or adverse effects were observed after autopsy, which proved the broad-spectrum safety of DC (121). Dandekar P et al. evaluated the toxicology of curcumin-loaded nanoparticles. The results of acute toxicity studies showed that the dose of 2000 mg/kg was non-toxic, and the subacute toxicity studies demonstrated the safety of long-term administration at the usual therapeutic dose of 100 mg/kg curcumin and twice the therapeutic dose (122).

### 4.7 The Strengths and Limitations

The strengths of this study is that to our knowledge, this is the first systematic review and meta-analysis of RCTs on the efficacy of turmeric and its curcumin on arthritis. The limitations of this study are: (1) Because curcumin has not been widely used in clinical practice, the sample size of the included studies is limited. (2) There may be omissions in the collection of documents and data extraction or the researcher’s subjective judgment is not strict. (3) The RCTs included in this study were at high risk of bias. The authors of some RCTs were funded by drug manufacturers or were employees, which may introduce bias. (4) Languages are limited to English and Chinese, and related studies in other languages are not included.

## 5 Conclusion

In summary, Curcumin and Curcuma longa Extract may improve symptoms and inflammation levels in people with arthritis. However, due to the low quality and small quantity of RCTs, the conclusions need to be interpreted carefully. Limited by the sample size of the included studies, large-sample, multi-center clinical trials are still needed for correction or verification.

## Data Availability Statement

The original contributions presented in the study are included in the article/**Supplementary Material**. Further inquiries can be directed to the corresponding authors.

## Author Contributions

TY and LZ contributed equally to this work. All authors contributed to the article and approved the submitted version.

## Conflict of Interest

The authors declare that the research was conducted in the absence of any commercial or financial relationships that could be construed as a potential conflict of interest.

## Publisher’s Note

All claims expressed in this article are solely those of the authors and do not necessarily represent those of their affiliated organizations, or those of the publisher, the editors and the reviewers. Any product that may be evaluated in this article, or claim that may be made by its manufacturer, is not guaranteed or endorsed by the publisher.
