# Supplementary material for: Efficacy and Safety of Curcumin and Curcuma longa Extract in the Treatment of Arthritis: A Systematic Review and Meta-Analysis of Randomized Controlled Trial
Source: Front Immunol. 2022 Jul 22;13:891822. doi: 10.3389/fimmu.2022.891822 (PMC9353077; doi:10.3389/fimmu.2022.891822)
Supplement: Supplementary file 1 [file Table_1.docx]

**Table S1.** Search Strategies for Pubmed and Embase

| **PubMed** | (Curcumin OR Curcumas OR Tumeric OR Tumerics OR Turmeric OR Turmerics OR Curcuma zedoaria OR Curcuma zedoarias OR zedoaria, Curcuma OR Zedoary zedoaria OR Zedoary zedoarias OR zedoaria, Zedoary OR Curcuma longa OR Curcuma longas OR longa, Curcuma OR Curcuma Longa)  AND  ((Rheumatoid arthritis OR Arthritis, Rheumatoid) OR (Osteoarthritis OR Osteoarthritides OR Osteoarthrosis OR Osteoarthroses OR Arthritis, Degenerative OR Arthritides, Degenerative OR Degenerative Arthritides OR Degenerative Arthritis OR Osteoarthrosis Deformans) OR (Spondyloarthritis Ankylopoietica OR Ankylosing Spondylarthritis OR Ankylosing Spondylarthritides OR Spondylarthritides, Ankylosing OR Spondylarthritis, Ankylosing OR Ankylosing Spondylitis OR Spondylarthritis Ankylopoietica OR Bechterew Disease OR Bechterew's Disease OR Bechterews Disease OR Marie-Struempell Disease OR Marie Struempell Disease OR Rheumatoid Spondylitis OR Spondylitis, Rheumatoid OR Spondylitis Ankylopoietica OR Ankylosing Spondyloarthritis OR Ankylosing Spondyloarthritides OR Spondyloarthritides, Ankylosing OR Spondyloarthritis, Ankylosing) OR (Juvenile Arthritis OR Arthritis, Juvenile Chronic OR Chronic Arthritis, Juvenile OR Juvenile Rheumatoid Arthritis OR Arthritis, Juvenile Idiopathic OR Juvenile Chronic Arthritis OR Arthritis, Juvenile Rheumatoid OR Rheumatoid Arthritis, Juvenile OR Juvenile Idiopathic Arthritis OR Idiopathic Arthritis, Juvenile OR Juvenile-Onset Still Disease OR Juvenile Onset Still Disease OR Still's Disease, Juvenile-Onset OR Juvenile-Onset Still's Disease OR Still's Disease, Juvenile Onset OR Still Disease, Juvenile-Onset OR Still Disease, Juvenile Onset OR Systemic Arthritis, Juvenile OR Arthritis, Juvenile Systemic OR Juvenile Systemic Arthritis OR Juvenile-Onset Stills Disease OR Juvenile Onset Stills Disease OR Stills Disease, Juvenile-Onset OR Polyarthritis, Juvenile, Rheumatoid Factor Positive OR Polyarthritis, Juvenile, Rheumatoid Factor Negative OR Oligoarthritis, Juvenile OR Juvenile Oligoarthritis OR Enthesitis-Related Arthritis, Juvenile OR Arthritis, Juvenile Enthesitis-Related OR Enthesitis Related Arthritis, Juvenile OR Juvenile Enthesitis-Related Arthritis OR Psoriatic Arthritis, Juvenile OR Arthritis, Juvenile Psoriatic) OR (Arthritis, Gouty OR Gouty Arthritis OR Arthritides, Gouty OR Gouty Arthritides) OR (Arthritides, Reactive OR Reactive Arthritides OR Reactive Arthritis OR Arthritis, Post-Infectious OR Arthritides, Post-Infectious OR Arthritis, Post Infectious OR Post-Infectious Arthritides OR Post-Infectious Arthritis OR Post Infectious Arthritis OR Postinfectious Arthritis OR Arthritis, Postinfectious OR Arthritides, Postinfectious OR Postinfectious Arthritides OR Reiter Syndrome OR Reiter's Disease OR Reiters Disease OR Reiter's Syndrome OR Reiters Syndrome OR Reiter Disease OR Arthritis, Reactive) OR (Infectious Arthritis OR Arthritis, Viral OR Viral Arthritis OR Arthritis, Bacterial OR Bacterial Arthritides OR Arthritis, Septic OR Septic Arthritis OR Arthritides, Bacterial OR Bacterial Arthritis OR Arthritis, Suppurative OR Suppurative Arthritis OR Arthritis, Infectious) OR (enteropathic arthritis OR traumatic arthritis) OR (Psoriasis, Arthritic OR Arthritic Psoriasis OR Psoriatic Arthritis OR Psoriasis Arthropathica OR Psoriatic Arthropathy OR Arthropathies, Psoriatic OR Arthropathy, Psoriatic OR Psoriatic Arthropathies OR Arthritis, Psoriatic) OR (Arthritis OR Arthritides OR Polyarthritis OR Polyarthritides))  AND  (random* controlled trial [pt] OR controlled clinical trial* [pt] OR randomized [tiab] OR placebo [tiab] OR drug therapy [sh] OR random* [tiab] OR trial* [tiab] OR group* [tiab])  NOT  (animals [mh] NOT humans [mh]) |
| --- | --- |
| **EMBASE** | 1 Curcumin/  2 Curcumas/  3 Tumeric/  4 Tumerics/  5 Turmeric/  6 Turmerics/  7 Zedoary zedoaria/  8 Zedoary zedoarias Curcuma longa/  9 Curcuma zedoaria/  10 Curcuma zedoarias/  11 Curcuma longas/  12 Curcuma Longa/  13 1-12/or  14 Osteoarthritis/exp  15 Osteoarthritides/  16 Osteoarthrosis/  17 Osteoarthroses/  18 Degenerative Arthritides/  19 Degenerative Arthritis/  20 Osteoarthrosis Deformans/  21 14-20/or  22 Spondyloarthritis Ankylopoietica/  23 Ankylosing Spondylarthritis/  24 Ankylosing Spondylarthritides/  25 Ankylosing Spondylitis/  26 Spondylarthritis Ankylopoietica/  27 Bechterew Disease/  28 Bechterews Disease/  29 Marie-Struempell Disease/  30 Marie Struempell Disease/  31 Rheumatoid Spondylitis/  32 Spondylitis Ankylopoietica/  33 Ankylosing Spondyloarthritis/  34 Ankylosing Spondyloarthritides/  35 22-34/or  36 Arthritis, rheumatoid/exp  37 Rheumatoid arthritis/  38 36-37/or  39 Arthritis, Juvenile/exp  40 Juvenile Arthritis/  41 Juvenile Rheumatoid Arthritis/  42 Juvenile Chronic Arthritis/  43 Juvenile Idiopathic Arthritis/  44 Juvenile-Onset Still Disease/  45 Juvenile Onset Still Disease/  46 Juvenile Systemic Arthritis/  47 Juvenile-Onset Stills Disease/  48 Juvenile Onset Stills Disease/  49 Juvenile Oligoarthritis/  50 39-49/or  51 Arthritis, Gouty/exp  52 Gouty Arthritis/  53 Gouty Arthritides/  54 51-53/or  55 Arthritides, Reactive/exp  56 Reactive Arthritides/  57 Reactive Arthritis/  58 Post-Infectious Arthritides/  59 Post-Infectious Arthritis/  60 Post Infectious Arthritis/  61 Postinfectious Arthritis/  62 Postinfectious Arthritides/  63 Reiter Syndrome/  64 Reiters Disease/  65 Reiters Syndrome/  66 Reiter Disease/  67 55-66/or  68 Arthritis, Infectious/exp  69 Infectious Arthritis/  70 Viral Arthritis/  71 Bacterial Arthritides/  72 Septic Arthritis/  73 Bacterial Arthritis/  74 Suppurative Arthritis/  75 68-74/or  76 enteropathic arthritis/  77 traumatic arthritis/  78 76-77/or  79 Arthritis, Psoriatic/exp  80 Arthritic Psoriasis/  81 Psoriatic Arthritis/  82 Psoriasis Arthropathica/  83 Psoriatic Arthropathy/  84 Psoriatic Arthropathies/  85 79-84/or  86 Arthritis/exp  87 Arthritides/  88 Polyarthritis/  89 Polyarthritides/  90 86-89/or  91 21 or 35 or 38 or 50 or 54 or 67 or 75 or 78 or 85 or 90  92 13 and 91  93 randomized controlled trial/  94 single blind procedure/  95 double blind procedure/  96 crossover procedure/  97 93-96/or  98 92 and 97 |
